# The coronavirus diseases 2019 (COVID-19) pneumonia with spontaneous pneumothorax: a case report

**DOI:** 10.1186/s12879-020-05384-x

**Published:** 2020-09-09

**Authors:** Xiaoxing Chen, Guqin Zhang, Yueting Tang, Zhiyong Peng, Huaqin Pan

**Affiliations:** 1 Department of Geriatrics, Renmin Hospital of Wuhan University, Wuhan University, Wuhan, China; 2 Department of Respiratory and Critical Care Medicine, Zhongnan Hospital of Wuhan University, Wuhan, 430071 China; 3 Department of Clinical Laboratory, Zhongnan Hospital of Wuhan University, Wuhan, 430071 China; 4 Department of Critical Care Medicine, Zhongnan Hospital of Wuhan University, 169 Eastlake Rd., Wuchang district, Wuhan, 430071 Hubei province China

**Keywords:** COVID-19, SARS-CoV-2 virus, Pneumonia, Pneumothorax

## Abstract

**Background:**

The outbreak of the novel coronavirus (COVID-19) that was firstly reported in Wuhan, China, with cases now confirmed in more than 100 countries. However, COVID-19 pneumonia with spontaneous pneumothorax is unknown.

**Case presentation:**

We reported a case of 66-year-old man infected with COVID-19, presenting with fever, cough and myalgia; The patient received supportive and empirical treatment including antiviral treatment, anti-inflammatory treatment, oxygen supply and inhalation therapy; The symptoms, CT images, laboratory results got improved after the treatments, and a throat swab was negative for COVID-19 PCR test; However, on the hospital day 30, the patient presented with a sudden chest pain and dyspnea. CT showed a 30–40% left-sided pneumothorax. Immediate thoracic closed drainage was performed and his dyspnea was rapidly improved. With five more times negative PCR tests for SARS-CoV-2 virus, the patient was discharged and home quarantine.

**Conclusion:**

This case highlights the importance for clinicians to pay attention to the appearance of spontaneous pneumothorax, especially patients with severe pulmonary damage for a long course, as well as the need for early image diagnose CT and effective treatment once pneumothorax occurs.

## Background

In late December 2019, an outbreak of the novel coronavirus (COVID-19) that was firstly reported in Wuhan, China, and was characterized as a pandemic by the WHO on March 11 [1, 2]. As of April 1, there were about 823,626 confirmed cases and 40,598 deaths worldwide [3]. Nowadays, the methods for the definitive diagnosis and treatment of patients with mild symptoms have been well established [4], however clinical manifestations, management and prognosis of COVID-19 pneumonia with complications such as pneumothorax may be much different. In this study, we report a case of COVID-19 pneumonia patient who developed spontaneous pneumothorax, which may provide further evidence for the suggestive management for such patients.

## Case presentation

**Fig. 1:**
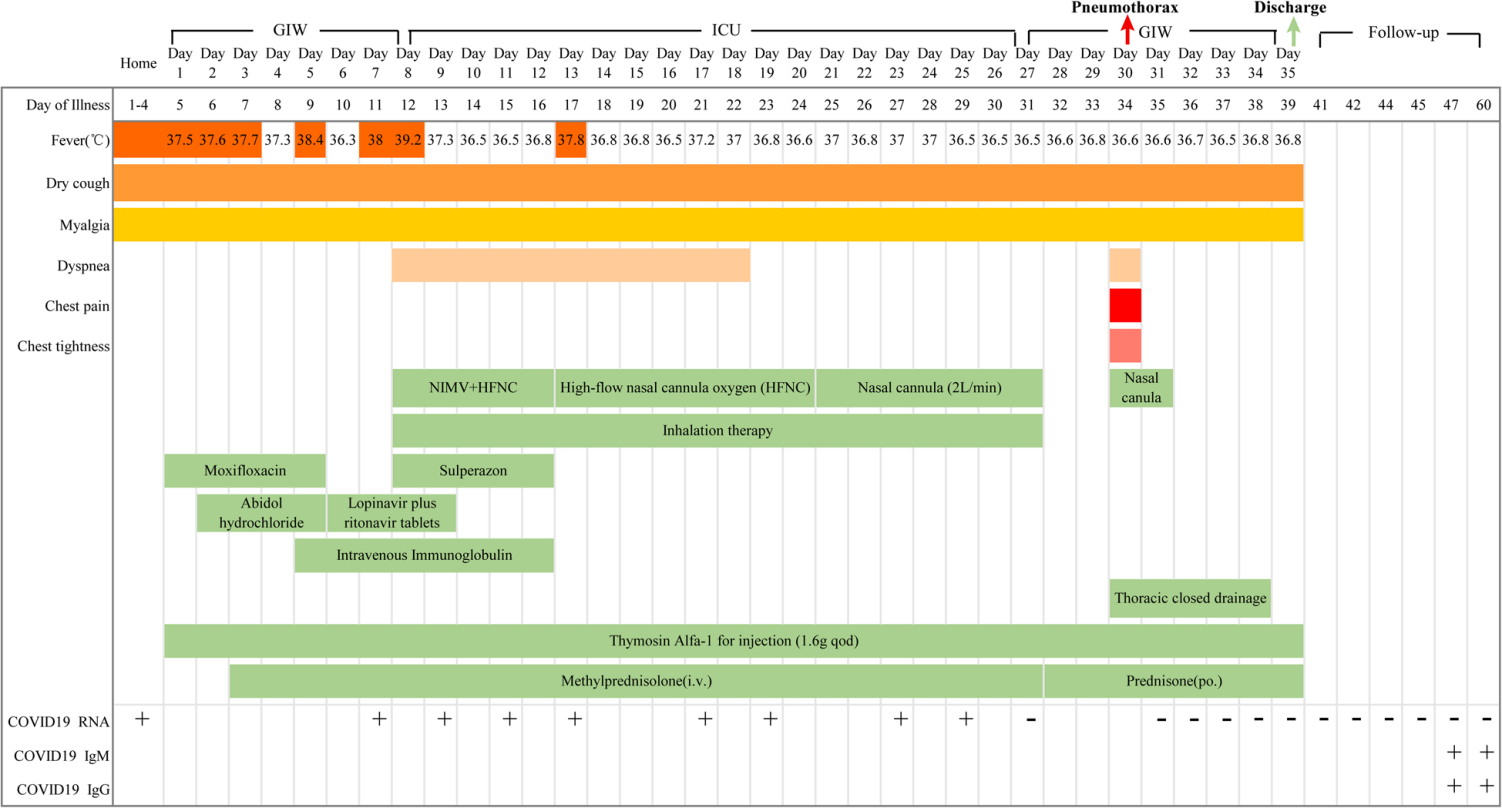
Timeline of disease course according to days from symptom onset, days from admission, and days of follow-up. from Jan 18-Mar 17, 2020. NIMV, non-invasive mechanical ventilation

**Fig. 2:**
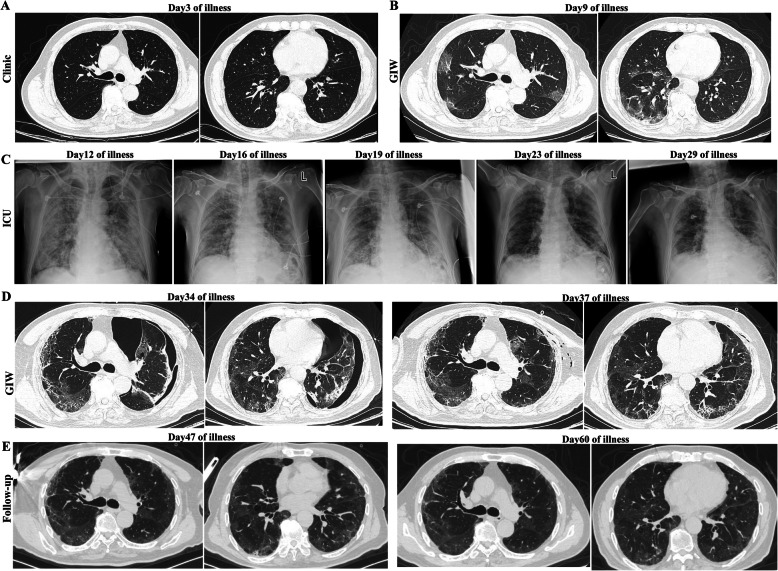
High-resolution computed tomography images during the disease course. **a**. ground-glass opacities (GGO) in the basal segment of the right lower lobe; **b**. multiple patchy ground-glass shadows in the lower lobe of both lungs. **c**. chest X-ray image before and after treatment in ICU. **d**. Emergent chest CT scan showed a 30–40% left-sided pneumothorax, and partially reexpansion of the left lung after treatment. **e**. gradual abortion of lung lesions during follow-up

The patient is a 66-year-old man living in Wuhan, who reported that he had an initial symptom of fever, dry cough and myalgia on January 18, without chills, dyspnea, chest pain, or diarrhea (Fig. 1). Two days later (January 20), he went to the clinic because of suspicious COVID-19 infection. The CT scan showed ground-glass opacities (GGO) in the basal segment of the right lower lobe ***(***Fig. 2***)***. Subsequently, a throat swab was obtained, and the patient was confirmed of COVID-19 infection by the reverse real-time PCR assay on January 21.On day 5 of illness onset, he was admitted to the general isolation ward (GIW) in Zhongnan Hospital of Wuhan University. The patient did not have a history of any underlying pulmonary disease, which were important on the incidence of spontaneous pneumothorax, such as COPD, cystic pulmonary fibrosis, interstitial lung disease, CTD or asthma; Physical examination revealed a body temperature of 36.3 °C, the blood pressure of 126/75 mmHg, a pulse of 71 beats per minute, the respiratory rate of 17 breaths per minute. Laboratory results were summarized as follows:

The lymphocyte count and percentage continuedly decreased (Table 1).

**Table 1:** Clinical laboratory results

Mearure	Normal range	1/23	1/26	1/28	1/29	1/31	2/1	2/2	2/3	2/4	2/5	2/6	2/7	2/8	2/9	2/10	2/12	2/14	2/15	2/16	2/19	3/4	3/17
		Day6	Day9	Day11	Day12	Day14	Day15	Day16	Day17	Day18	Day19	Day20	Day21	Day22	Day23	Day24	Day26	Day28	Day29	Day30	Day33	Day47	Day60
WBC(× 10^9^)	3.5–9.5	2.72	3.85	6.88	8.67	7.01	5.69	7.3		8.84	10.5	10.72	10.26	12.96	13.42	13.42	13.42	11.15			8.65	8.6	7.83
Hgb (g/L)	130–175	144.6	142.4	134.9	143.4	135.7	124.9	129.8		138.2	135.2	141	141.4	126	134.1	118	120	121.3			127.5	124.4	124.8
LYM(%)	20–50	19	14	4.3	0.5	2.3	3.2	4.1		3	93.4	2.6	90.9	2	4.6	2	2.1	3.7			10.9	23.7	15.6
NEUT(%)	40–75	64.6	72.4	90.5	95.6	92.5	91.6	92.6		94.8	93.6	93.6	5	95.2	92.1	96.2	95.8	90.6			78.8	65.8	66.4
LYM(×10^9^)	1.1–3.2	0.52	0.54	0.3	0.04	0.16	0.18	0.3		0.26	0.36	0.28	0.51	0.26	0.62	0.27	0.28	0.41			0.94	2.04	1.22
NEUT(×10^9^)	1.8–6.3	1.76	2.79	6.22	8.29	6.49	5.21	6.76		8.38	9.84	10.04	9.32	12.33	12.35	12.9	12.85	10.1			6.82	5.68	5.2
PCT (ng/mL)	< 0.05	< 0.05	< 0.05	< 0.05	0.46	0.61	0.3	0.24		0.3	0.19	0.09	0.05		< 0.05						< 0.05		0.05
cTNI (pg/ml)	0–26.2				398.7	43.5	20.9												1.7				
MYO (ng/ml)	< 140.1				321.1	59.3	45.8																
CKMB (ng/ml)	0–6.6				1.9	2.1	1.2																
NT-ProBNP (pg/ml)	< 100				2200	644	496					90.1		40.1					< 10				
ALT(U/L)	9–50	19		30					60						110				55		54	32	21
AST(U/L)	15–40	20		26					72						31				19		20	14	16
TP(g/L)	65–85	60.4		59.1					67.5						57				58		57	61.7	61
ALB(g/L)	40–55	36.2		30.4					33.5						27.8				33.8		33.2	37.7	37.2
BUN (mmol/L)	2.8–7.6	6.01		6.84					5.6						7.56				6.61		6.91	7.37	4.38
CREA (ummol/L)	64–104	70		72					44.8						72.4				54.7		57.4	53.1	55.8
D-dimer (ng/ml)	0–500	141						8276				8458							3316		3912		
PH	7.35–7.45				7.503	7.422	7.398	7.44	7.484	7.447	7.413	7.41	7.46	7.438	7.368	7.402	7.374		7.468	7.373			
PCO2(mmHg)	35–45				23.9	40	40.5	40.3	40.1	32.9	35.5	29.7	33.3	34.4	42.8	39.8	42.5		46	50.8			
SpO2(%)	95–98%				93	96.4	94.2	90.7	90.5	92.7	91.6	95.4	94.6	90.4	93.3	93.7	97.8		96.7	98.3			
P/F (mmHg)	400–500				60.2	63.3	81.4	80	71	95.1	93.8	175.8	153.1	127.8	238.3	191.7	273.7		383.8	343.3			

*WBC* white blood cell, *Hgb* hemoglobin, *NEUT* neutrophil, *LYM* lymphocyte, *PCT* procalcitonin, *cTNI* cardiac troponin I, *MYO* myoglobin, *CKMB* Creatine kinase-MB, *ALT* alanine aminotransferase, *AST* aspartate aminotransferase, *TP* total protein, *GLB* albumin, *BUN* blood urea nitrogen, *CREA* serum creatinine, *PCO2* Partial Pressure of Carbon Dioxide, *P/F* Partial Pressure of arterial oxygen /fraction of inspiratory oxygen

The neutrophils were initially normal but elevated on day 11 of illness onset (Table 1).

The level of serum procalcitonin was normal (Table 1).

The hepatic function measures were normal (Table 1).

The follow-up CT scan (day 9 of illness) showed multiple patchy ground-glass shadows in the lower lobe of both lungs, which indicated the progression.

The treatment in GIW was basically supportive and empirical. He was given lopinavir plus ritonavir (500 mg twice daily, po.) and abidol hydrochloride (200 mg three times daily, po.) as antiviral therapy, and moxifloxacin (400 mg once daily, i.v.) to prevent secondary infection. To attenuate lung inflammation, low dose of methylprednisolone (40 mg once daily, i.v.) and intravenous immunoglobulin (20 g once daily for 5 days, i.v.) was administered.

The patient’s symptoms continued unabated. On the day 12 of illness, the patient suddenly developed dyspnea with a higher fever of 39.2 °C and a decreased oxygen saturation value of 80%. He was immediately transferred to the Intensive Care Unit (ICU) and received discontinued non-invasive mechanical ventilation (NIMV) plus high-flow nasal cannula (HFNC) oxygen therapy. The initial FiO2 for HFNC was 100%, and maintained with a gas flow-rate of 50 L/min. A model of continuous positive airway pressure-pressure support ventilation (CPAP/PSV) for NIMV was intermittently conducted with an adjustable 5-12cmH_2_O positive end expiratory pressure (PEEP). The methylprednisolone dose was elevated to 80 mg every 12 h, and intravenous immunoglobulin was administered for five days. Given the increased neutrophils and the procalcitonin level as shown in Table 1, we started the treatment with cefoperazone sulbactam sodium (3 g every 8 h, i.v.). Besides, inhalation therapy (Budesonide Suspension 1 mg, Ipratropium Bromide Solution 500μg plus Salbutamol Sulfate 5 mg, every 6 h, inh.) was given to dilate bronchioles. After receiving medications, the patient’s oxygen saturation value increased to 94%. Laboratory results were listed in Table 1. On the day 17 of illness, the patient’s clinical condition improved and received HFNC therapy without NIMV. The methylprednisolone was gradually decreased to 20 mg twice daily, and the supplemental oxygen delivered by nasal cannula at 2 l per minute was started on day 25 of illness, which maintained the oxygen saturation value above 96%. On day 31 of illness, a throat swab was negative for COVID-19 PCR test. Chest X-ray showed diffuse patchy shadows in both lungs, but the shadows were improved. Hence, the patient was transferred to GIW.

In GIW, methylprednisolone was discontinued and prednisone (20 mg twice daily, orally) was administered for anti-inflammatory treatment. Supplemental oxygen was discontinued, and his oxygen saturation value maintained above 94% when he was breathing ambient air. On the hospital day 30, the patient presented with a sudden chest pain, with dyspnea and chest tightness. Emergent chest CT showed a 30–40% left-sided pneumothorax. Immediate thoracic closed drainage was performed and his dyspnea was rapidly improved. The supplemental oxygen was delivered by nasal cannula at 2 l per minute. Subsequent CT showed partially reexpansion of the left lung, with little free air in the left thorax. There were multiple patchy ground-glass density pulmonary infiltrates and fibrosis in both lungs. The chest tube was extracted on the hospital day 34 (day 38 of illness). The patient remained afebrile for more than twenty days, and all symptoms have resolved except myalgia, which was decreased in severity. With five more times negative PCR tests for SARS-CoV-2 virus, the patient was discharged and home quarantine.

## Discussion and conclusion

Latest studies have revealed that the COVID-19 shares over 88% homology with two bat-derived severe acute respiratory syndrome (SARS)-related coronaviruses [5]. Research for SARS outbreak in 2003 demonstrated that spontaneous pneumothorax is complicated with a rate of 1.7% in critically ill cases [6].. Severe pulmonary lesions may predispose to spontaneous pneumothorax. Without timely management the pneumothorax can be fatal. This means that manifestations and treatment methods of COVID-19 pneumonia with pneumothorax require careful consideration.

There are already several case reports about this pneumothorax related to COVID-19 [7–9], however they were not presented in recovery time. In this case, the patient did not have a history of any underlying pulmonary disease. Laboratory examinations, symptoms and chest CT were similar to general population without pneumothorax. The onset of the pneumothorax of this patient occurred on the day 29 after the initial diagnosis of COVID-19, which suggested that a sustained period of extensive lung injury may increase susceptibility of pneumothorax. It is crucial for clinicians to pay attention to the appearance of spontaneous pneumothorax which sometimes would occur unpredictably in delayed feature, especially patients with severe pulmonary damage for a long course. Given symptoms may be subtle, respiratory monitor and early CT scan can be of a great benefit. Secondly, this case highlights the importance for immediate and active treatment [10]. In the present case, we mainly adopted thoracic closed drainage and offered supplemental oxygen. What’s more, the follow-up CT is needed to observe the conversion of pulmonary damage and to avoid recurrence of the pneumothorax.

This is a first report of a COVID-19 pneumonia patient with spontaneous pneumothorax in Wuhan, which illustrates several aspects including the clinical features and therapeutic course. Following an active treatment regimen consisting of thoracic closed drainage and supplemental oxygen therapy, the patient recovered well. However, because of the limited case, predisposing factors, onset time of COVID-19 pneumonia patient with spontaneous pneumothorax occurring, and treatment protocol deserve further research.

## Data Availability

All data generated or analyzed during this study are included in the published article.

## Abbreviations

COVID-19: Coronavirus disease 2019
SARS-COV-2: Severe acute respiratory syndrome coronavirus 2
CT: Computed tomography
GGO: Ground-glass opacities
GIW: General isolation ward
ICU: Intensive Care Unit
NIMV: Non-invasive mechanical ventilation
HFNC: High-flow nasal cannula
CPAP/PSV: Continuous positive airway pressure-pressure support ventilation
PEEP: Positive end expiratory pressure
